# Imaging Chemical Kinetics of Radical Polymerization with an Ultrafast Coherent Raman Microscope

**DOI:** 10.1002/advs.201903644

**Published:** 2020-03-09

**Authors:** Haozheng Li, Yong Cheng, Huajun Tang, Yali Bi, Yage Chen, Guang Yang, Shoujing Guo, Sidan Tian, Jiangshan Liao, Xiaohua Lv, Shaoqun Zeng, Mingqiang Zhu, Chenjie Xu, Ji‐Xin Cheng, Ping Wang

**Affiliations:** ^1^ Britton Chance Center for Biomedical Photonics Wuhan National Laboratory for Optoelectronics Huazhong University of Science and Technology Wuhan Hubei 430074 China; ^2^ MoE Key Laboratory for Biomedical Photonics Collaborative Innovation Center for Biomedical Engineering School of Engineering Sciences Huazhong University of Science and Technology Wuhan Hubei 430074 China; ^3^ School of Chemical and Biomedical Engineering Nanyang Technological University Singapore 637457 Singapore; ^4^ Department of Biomedical Engineering Boston University Boston MA 02215 USA

**Keywords:** chemical kinetics, polymer synthesis, radical polymerization, stimulated Raman scattering, ultrafast chemical imaging, vibrational spectroscopy

## Abstract

Numerous mechanisms have been proposed for polymerization to provide qualitative and quantitative prediction of how monomers spatially and temporally arrange into the polymeric chains. However, less is known about this process at the molecular level because the ultrafast chemical reaction is inaccessible for any form of microscope so far. Here, to address this unmet challenge, a stimulated Raman scattering microscope based on collinear multiple beams (COMB‐SRS) is demonstrated, which allows label‐free molecular imaging of polymer synthesis in action at speed of 2000 frames per second. The field of view of the developed 2 kHz SRS microscope is 30 × 28 µm^2^ with 50 × 46 pixels and 7 µs dwell time. By catching up the speed of chemical reaction, COMB‐SRS is able to quantitatively visualize the ultrafast dynamics of molecular vibrations with submicron spatial resolution and sub‐millisecond temporal resolution. The propagating polymer waves driven by reaction rate and persistent UV initiation are observed in situ. This methodology is expected to permit the development of novel functional polymers, controllable photoresists, 3D printing, and other new polymerization technologies.

## Graphical abstract

An ultrafast collinear multiple beam‐based stimulated Raman scattering microscope is developed to achieve direct imaging and quantification of polymerization kinetics in situ. By label‐free and molecular imaging of the polymerization process in action at a frame rate of 2 kHz, the polymer wave is observed, which is actually driven by radicals persistently generated in the UV initiation center.

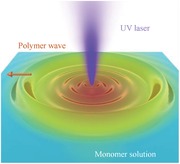

## 1 Introduction

Polymerization reaction has made considerable impact on different aspects of chemistry. A basic understanding of polymerization processes contributes to the knowledge of process development and the improvement in the structures and properties of the polymer networks. Conventionally, this has been studied using technologies such as NMR spectroscopy,^[^ ^1^ ^]^ mass spectrometry,^[^ ^2^ ^]^ electronic microscopy,^[^ ^3^ ^]^ IR spectroscopy,^[^ ^4^ ^]^ and chromatography.^[^ ^5^ ^]^ In a recent advance, fluorescent labeling of monomers with aggregation‐induced emission dyes permits the imaging of aggregation and viscosity change upon polymerization.^[^ ^6^ ^]^ However, all these approaches are limited to regular characterizations of monomers or polymers in bulk, and only provide insight into the microscopic level with poor spatial and temporal resolution. Label‐free Raman spectroscopy can chemically identify and quantify monomer and polymer with linear signals to their concentrations.^[^ ^7^ ^]^ However, owing to extremely weak Raman scattering photons, insensitive Raman microscopy is too slow for imaging of polymerization reactions.

Based on optical nonlinear effect, both coherent anti‐Stokes Raman scattering (CARS)^[^ ^8^ ^]^ and stimulated Raman scattering (SRS)^[^ ^9^ ^]^ microscopies reached chemical imaging at video‐rate speed.^[9c,10]^ To resolve spatially and spectrally overlapping chemical compounds, multiplex and hyperspectral SRS microscopy has been developed with high spatial resolution (i.e., 0.1–0.5 µm).^[9c,11]^ In addition, CARS and SRS microscopes have been extensively used to study polymers, polymer blends,^[^ ^12^ ^]^ lignin,^[^ ^13^ ^]^ biomass processing,^[^ ^14^ ^]^ and anion depletion.^[^ ^15^ ^]^ Nevertheless, the frame rate of them is still far from capturing the ultrafast processes of chemical reactions, which typically happen in millisecond scale. Because of the lack of online and fast monitoring instruments, the radical polymerization reactions remain largely inaccessible for microscopic imaging and dynamic characterization so far.

In this study, we demonstrate label‐free chemical imaging of radical polymerization process, including initiation, propagating, and termination dynamics by pushing the speed of SRS microscopy to an unprecedented level of 2000 frames per second. Specifically, we developed a collinear multiple beam‐based SRS microscope (COMB‐SRS), in which two acousto‐optic deflectors (AOD) were implemented in pump and Stokes lasers to realize SRS comb. By catching up the speed of active radical polymerization, we tracked the instantaneous kinetics of polymerization process and the dynamics of controlled radical propagation with submicron spatial resolution and sub‐millisecond temporal resolution. We present the observation of propagating polymer waves in real time and showed that such waves were driven by reaction rate and persistent supply of free radicals from the initiation center.

## 2 Results

### 2.1 Characterization of Polyacrylamide Synthesis by SRS Spectra

**Figure 1:**
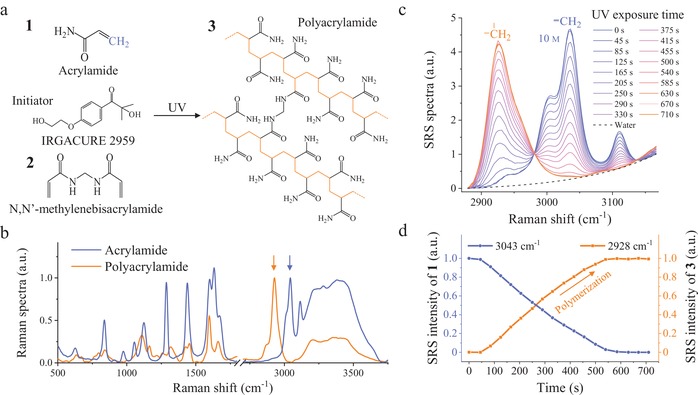
Polymerization process of hydrogel characterized by SRS spectra. a) Schematic of UV laser‐induced hydrogel polymerization. b) Raman spectra of acrylamide and polyacrylamide. c) SRS spectra recording the polymerization process. d) Chemical quantitation of polymer conversion by measuring the two distinct CH_2_ vibrations.

We explored the polymerization process of acrylamide (**1**) under the presence of the cross‐linking agent *N*,*N*′‐methylenebisacrylamide (**2**). This hydrogel polymerization process experiences three distinct phases, including chemical initiation, radical propagation, and termination as typical polymers (**Figure** **1**a). First, the initiator (e.g., Irgacure 2959) is decomposed to free radicals by activation energy from UV photon (Figure S1, Supporting Information). Second, the formed radical functions to break the weak carbon–carbon double bond (C=C) in the first monomer, and therefore transfers the radical to this monomer. During the radical propagation, the polyacrylamide (**3**) forms by repeated addition of new monomer molecule to the radical chain. In the final termination step, the growing radical polymerization will cease to progress and end up by combination with another free radical or active polymer. In Figure 1b, we acquired spontaneous Raman spectra of both acrylamide and polyacrylamide, and found that their Raman spectra are significantly different from each other. Especially in C—H region, the acrylamide displays a characteristic Raman peak at 3043 cm^‐1^, which corresponds to the symmetric stretching of =CH_2_ (colored in blue in Figure 1a,b). Meanwhile, the clear Raman band on the left shoulder at 3004 cm^‐1^ is from HC= , and the Raman peak on the right at 3111 cm^‐1^ is assigned to asymmetric stretching of =CH_2_. In addition, the water in hydrogel shows broad O—H vibrational band around 3400 cm^‐1^. In contrast to the monomer, the major Raman band of polyacrylamide shifts to 2928 cm^‐1^, because the structures —HC=CH_2_ in monomers are opened to form periodical —HC—CH_2_—CH—CH_2_—CH— chain in the resulting polymer.^[^ ^16^ ^]^ Thus, the Raman spectra allow chemical identification of monomer and polymer by their distinct molecular vibrations.

Since the SRS spectra are identical to their corresponding spontaneous Raman spectra,^[9a]^ we performed fast hyperspectral SRS imaging^[11e]^ of hydrogel droplet exposed to steady UV illumination via a 360 nm lamp (schematic SRS setup in Figure S2 and shot noise limited detection in Figure S3, Supporting Information).^[^ ^17^ ^]^ Before the experiment, we validated that the near‐infrared lasers applied in SRS imaging have not interfered the polymerization process of hydrogel (Figure S4, Supporting Information). In Figure 1c, we recorded SRS spectra of gradually curing acrylamide solution (10 m) about every 40 s until the complete polymerization occurred after ≈700 s. As an encouraging result, we quantitatively observed Raman bands transferring from 3043 cm^‐1^ of monomer to 2928 cm^‐1^ of polymer. In Figure 1d, we chemically quantified the polymerization conversion by plotting normalized SRS intensities of these two Raman peaks versus UV exposure time (after subtraction of water background). In such slow polymerization process, the concentration of monomers decreased almost linearly with time, and that of resulting polymers grew inversely. Therefore, the SRS spectra precisely quantified one‐to‐one chemical conversion between =CH_2_ and —CH_2_ during photopolymerization, and proved great capability of online monitoring of chemical reactions.

### 2.2 Hyperspectral SRS Imaging of Active Polymer Synthesis

**Figure 2:**
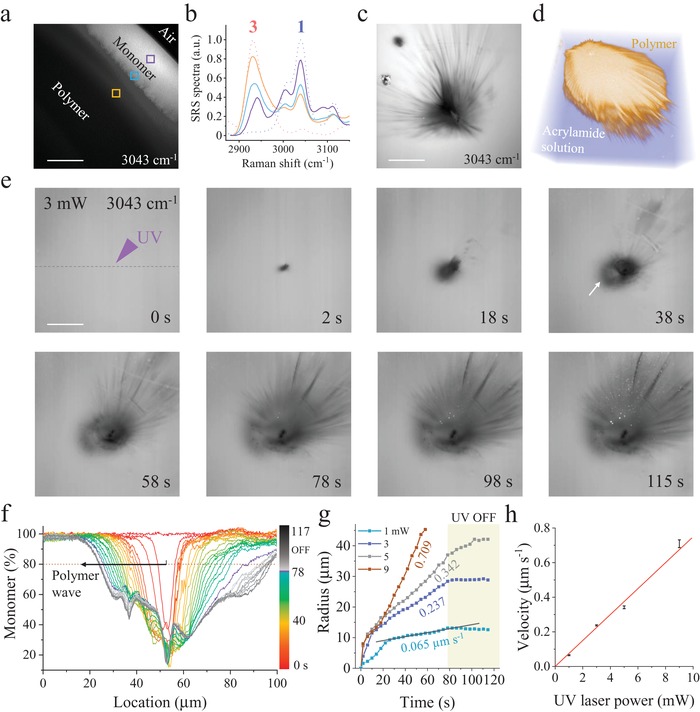
SRS imaging and quantification of polymer wave propagation. a) Hyperspectral SRS imaging of acrylamide/air interface after 3 min of uniform UV exposure. Scale bar, 50 µm. b) SRS spectra on the indicated locations in panel (a). The blue and orange dotted lines represent the SRS spectra of pure monomer (**1**) and polymer (**3**) for reference. c) Spectral SRS imaging of polymer formed by tightly focused UV laser. Scale bar, 30 µm. d) Two‐species 3D SRS imaging of both monomer and formed polymer structure. e) SRS recording of fast polymerization dynamics at every 4 s (Video S2, Supporting Information). Arrow indicates the polymer wave. Scale bar, 25 µm. f) Intensity profiles of monomer distribution across the initiation center (indicated by the dashed line in panel (e)). Purple curve indicates the boundary of UV laser on and off. g) Radius of propagating polymer waves with time at UV laser power of 1, 3, 5, and 9 mW. h) Linear dependence of propagation speeds of polymer waves with the UV laser power.

We further performed hyperspectral SRS mapping of the interface between hydrogel droplet and air after the sample was exposed with steady 395 nm UV illumination for 3 min (**Figure** **2**a). In Figure 2b, we illustrated SRS spectra on the indicated locations in Figure 2a, and found that the polymers bearing distinct Raman band at 2928 cm^‐1^ were more prone to form in the center of the droplet. Rather, the monomers remained largely in the peripheral region of the droplet, which appeared with great similarity to the features caused by coffee ring effect (concentration map; intensity profile in Figure S5, Supporting Information). In special cases, such as 3D microprinting^[^ ^18^ ^]^ and photo etching,^[^ ^19^ ^]^ the radical initiation required tightly focused UV laser to achieve controllable polymerization with high spatial resolution. Thus, we switched large‐area UV lamp to 396 nm laser, which was focused by a high NA objective on a droplet of acrylamide solution for 30 s. As shown in Figure 2c, the formed polymer exhibited apparent inhomogeneity in space. Importantly, the feather‐like structures that appeared with rich stochastic branches suggest that the radical chain is prone to remain growing along a specific direction until polymerization termination (concentration map in Figure S6; repeated data in Figure S7, Supporting Information). In fact, such structures were not simply flat in 2D plane, but actually grew and stretched out as acicular hydrogel cured in 3D space (Figure 2d and Video S1, Supporting Information). Such spatial inhomogeneity is sure to degrade the spatial resolution of 3D printing and other applications dramatically.

To monitor polymerization process with time, we further performed SRS imaging every 4 s immediately after the laser initiation (all images in Video S2, Supporting Information). As shown in Figure 2e, we clearly observed the forming polymers at initiation center even after 2 s. With continuous exposure, the spreading polymers grew outwards quickly. After 18 s, we began to spot apparent spiny structures, which rushed far out into the surrounding acrylamide solution. In the following time, these fast growing branches became stronger or wider in diameter. Unexpectedly, we also visualized another type of slower spreading polymer, which propagated homogeneously in all directions with time (indicated by white arrow). In Figure 2f, we plotted the profiles of monomer distribution across the initiation center (indicated by the dashed line in first image of Figure 2e), which quantitatively reflected the formation and propagation process of polymer with UV exposure time. At the first 2 s, the profile showed smooth Gaussian distribution of the disappeared monomers, and ≈60% of monomers were converted to polymer at the initiation center (Figure 2f, red curve). In the following polymerization evolution for 76 s, the chain reaction propagated to the periphery of initiation center at a steady speed (indicated by black arrow in Figure 2f). We intentionally turned off the UV laser at 78 s, and found that the polymerization almost stopped propagation after UV laser termination. Disregarding the interference of the acicular polymerization on the right part, we plotted the radius of spreading polymer with time to quantify the real dynamics of polymerization (Figure 2g, blue curve). We found that the polymerization process experienced two distinct phases. In the first phase about 10 s, the radius of the polymer increased very rapidly. In the second phase, the polymer began to propagate at a constant speed (i.e., slope of radius vs time). To confirm our observation, we further tuned the laser power of UV initiation from 1 to 3, 5, and 9 mW, and recorded the corresponding polymerization processes (Video S2, Supporting Information). As shown in Figure 2g, all polymerization processes driven at different laser power experienced similar two major dynamic phases. In the second phase, the propagation speeds of polymers were quantified by linear fitting to be 0.065, 0.237, 0.342, and 0.709 µm s^−1^, respectively. Unexpectedly, we discovered that the speed of polymer is actually proportional to the laser power of the applied UV laser (Figure 2h). In addition, as we turned off the UV laser, the propagation stopped or slowed down immediately. It implies that the polymer propagation is strictly correlated with the radical production from initiation center, which maintains persistent polymerization synthesis.

### 2.3 Ultrafast COMB‐SRS Imaging of Polymer Wave Propagation

**Figure 3:**
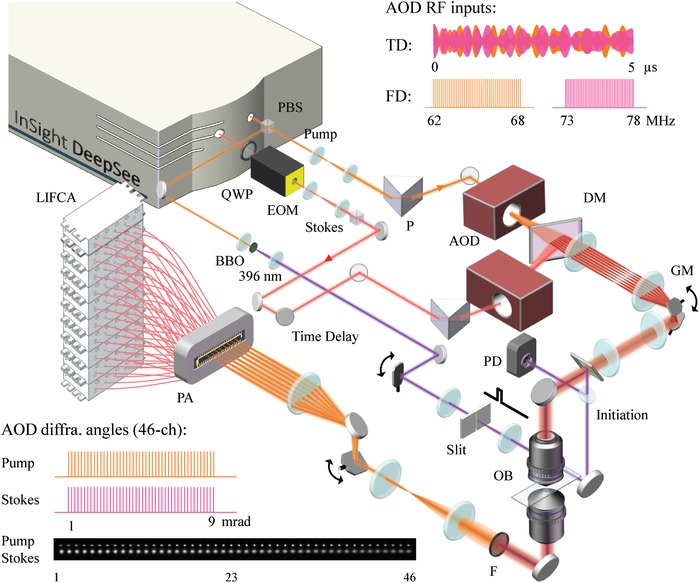
Schematic of the COMB‐SRS microscope. Upper‐right inset: RF waveform inputs to two AODs for generation of laser combs. FD, frequency domain; TD, time domain. Bottom‐left inset: diffraction angles and picture of 46 laser beam focuses. The pump and Stokes combs were separated intentionally for better illustration. AOD, acousto‐optic deflector; BBO, barium boron oxide; DM, dichroic mirror; EOM, electro‐optical modulator; F, filters; GM, galvanometer; LIFCA, lock‐in free circuits array; OB, objective; P, prism; PA, photodiode array; PBS, polarization beam splitter; PD, photodiode; QWP, quarter wave plate.

To understand how and when the polymer propagation formed, we explored the polymerization process with sub‐millisecond interval in a much shorter time scale, such as 1 s after initiation. Thus, we developed single‐frequency COMB‐SRS microscope with molecular imaging speed at 2000 frames per second (**Figure** **3**). Since the SRS signal only efficiently occurs at tightly focused point of the pulse laser, we utilized laser focus array to achieve COMB‐SRS working in parallel scanning mode. The setup of the entire instrument is challenging, and mainly consists of the following three subsystems. 1) The COMB‐SRS system specially equipped with two broadband AODs for pump and Stokes lasers (Figure S8, Supporting Information).^[^ ^20^ ^]^ Both AODs were independently fed with 46‐channel RF‐driving frequencies (upper‐right inset of Figure 3), which simultaneously generated 46 Gaussian laser beams with evenly separated diffraction angles (multiple first diffraction orders generated by 46 RF frequencies, bottom‐left inset of Figure 3 and Note S1, Supporting Information). Especially, two prisms were applied to compensate the spatial and temporal dispersion of AODs (Figure S9 and Note S2, Supporting Information).^[^ ^21^ ^]^ After dichroic mirror, we collinearly matched the 46 “fingers” in pump and Stokes laser combs by precisely adjusting the diffraction angles between them (fine‐tuning of the RF frequencies). After successful beam matching and a 4‐f conjugation system, we scanned the SRS comb by a 1 kHz galvanometer in vertical direction. With 60× high NA objective, we produced an array of laser focuses on the sample (i.e., picture in bottom‐left inset of Figure 3, a 300 mm lens was applied for illustration of the focus array), which enabled 2 kHz COMB‐SRS imaging. For all COMB‐SRS imaging process, the field of view (FOV) was 30 × 28 µm^2^ with 50 × 46 pixels, and the dwell time was 7 µs. The laser power for each pump (791 nm) and Stokes beam in the comb was about 7 mW. 2) In the detection side, we implemented a 46‐channel photodiode array to obtain SRS signal carried in each pump beam. To compensate the laser scanning and to restore the stationary laser comb for detection, we performed descanning by a 1 kHz galvanometer with opposite phase. In contrast to typical SRS microscope requiring expensive lock‐in amplifier to demodulate the weak SRS signal, our ultrafast SRS system adopted 46‐channel lock‐in‐free circuits array (LIFCA) at 10.5 MHz to demodulate multiple SRS signals from 46 pump beams (Figure S10 and Note S3, Supporting Information).^[^ ^22^ ^]^ For data acquisition, we employed three 16‐channel analog acquisition cards to transfer SRS images to computer at 2000 frames per second. 3) To introduce 396 nm UV laser to the system for precise polymerization initiation, we implemented a barium boron oxide (BBO) to double the laser frequency of a 791 nm laser (laser path colored in purple in Figure 3). Then, a triggered galvanometer turned the laser on or off with sub‐millisecond resolution by positioning the focused UV beam through a slit. More details of COMB‐SRS system are discussed in Experimental Section.

### 2.4 Ultrafast Imaging of Polymer Wave Driven by Transporting Reaction Rate

**Figure 4:**
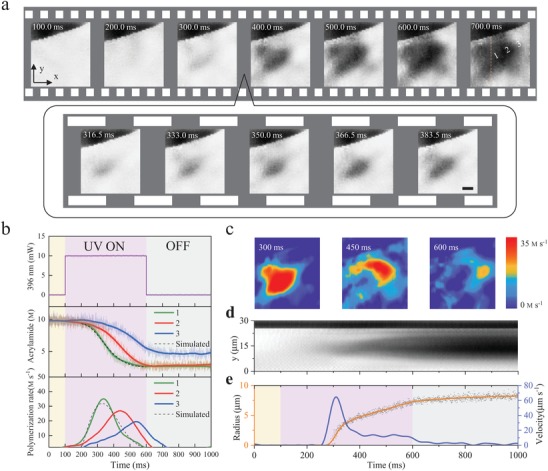
COMB‐SRS imaging of ultrafast dynamics of polymer wave propagation. a) Label‐free visualization of polymerization process with imaging speed of 2000 frames per second at 3043 cm^‐1^ (Video S3, Supporting Information). Only selected images are shown. The distances between 1, 2, and 3 are 6.38 and 4.32 µm, respectively. Scale bar, 5 µm. b) Dynamics of acrylamide concentration and polymerization rate on the indicated locations in panel (a). The colored regions show the time of UV laser on or off. c) Map of polymerization rate versus time (complete dynamics shown in Video S4, Supporting Information). d) Intensity profile of polymer forming with time (along the dashed line) in panel (a). e) Calculated radius (yellow) and propagation velocity (blue) of forming polymer with time. All data of radius were determined by 20% conversion of monomer to polymer.

As shown in **Figure** **4**a, we performed ultrafast SRS imaging of initiation process of polymerization in which 2000 images were recorded with time resolution of 0.5 ms in the first phase (all images in Video S3; image data processing in Figure S11, Supporting Information). After the waiting time for the first 100 ms, 10 mW UV laser was tuned on for 500 ms, and then turned off for the rest 400 ms (Figure 4b). Figure 4a displays SRS images between 300 and 400 ms, during which the polymerization process experienced a rapid change. As shown in Figure 4b, we plotted the concentrations of monomers with time on the selected locations (indicated by 1, 2, and 3 in Figure 4a). Even at the initiation center 1, the polymerization process did not respond immediately to the UV laser, but began to speed up almost after 100 ms (*t =* 200 ms) from initiation. On the locations of 2 and 3, the polymerization process delayed sequentially. By time differential of the concentration maps, we further revealed the spatial and temporal dynamics of polymerization rate (Figure 4c). Significantly, we found that the locations with maximum rate of polymerization were actually propagating to the peripheral space with time as polymer wave, which is beyond the current knowledge. For instance, on the initiation center 1, the polymerization rate (monomer disappearance rate) reached the maximum speed of about 35 m s^‐1^ after 236 ms, and slowed down afterward (bottom of Figure 4b). To model the process, we assumed that the polymerization rate is proportional to the concentrations of both monomers (*M*) and radicals (*R*). In this case, the radicals were persistently generated by UV laser in the initiation center, and therefore the concentration of radicals correlated proportionally with both time and laser power.^[^ ^23^ ^]^ Thus, we simulated the polymerization rate with formula: $\frac{dM}{dt}\;\propto \;MR\;\propto \;kMt$, and then derived correlation model of monomer concentration and polymerization rate with time: $M\;=\;a\;\times \;e^{-\frac{kt^{2}}{2}}+\;b;\;\dot{M}=\;-a\;\times \;k\;\times \;t\;\times \;e^{-\frac{kt^{2}}{2}}$. Here, the coefficient *k* is proportional to the UV laser power, and *a*, *b* are coefficients for simulation in Figure 4b (dotted curves). The observed process was well consistent with the estimates made by this model. Importantly, we experimentally visualized such chemical reaction wave relocated to position 2 after 105 ms (*t =* 205, traveled 6.38 µm) and further position 3 after 201 ms (total 10.7 µm). More specifically, the observed wave of polymerization rate was transporting outwards with time in 2D space (Figure 4c, Video S4, Supporting Information), which was driven by persistent production of radicals in the initiation center and higher concentration of monomer at the periphery. In Figure 4d, we further depicted the profile of polymer forming with the time (along yellow dashed line across the initiation center in Figure 4a), and thus measured the radius and propagation velocity of the polymer wave in Figure 4e. We calculated the polymerization velocity was as fast as ≈13 µm s^−1^ at the time of 400 ms until the UV laser was turned off at 600 ms. We further tested whether the polymerization will return after we turned on the UV laser initiation again. As we observed in Figure S12, Supporting Information, the polymerization process restored the previous growing trend as that when stopped. Overall, we observed the continuous polymer wave propagating in space, which was correlated with polymerization rate and driven by free radicals supplied by persistent laser initiation. Actually, such polymerization wave is also the nature of the phenomenon we visualized in Figure 2, where the polymer wave entered a very steady propagation mode.

**Figure 5:**
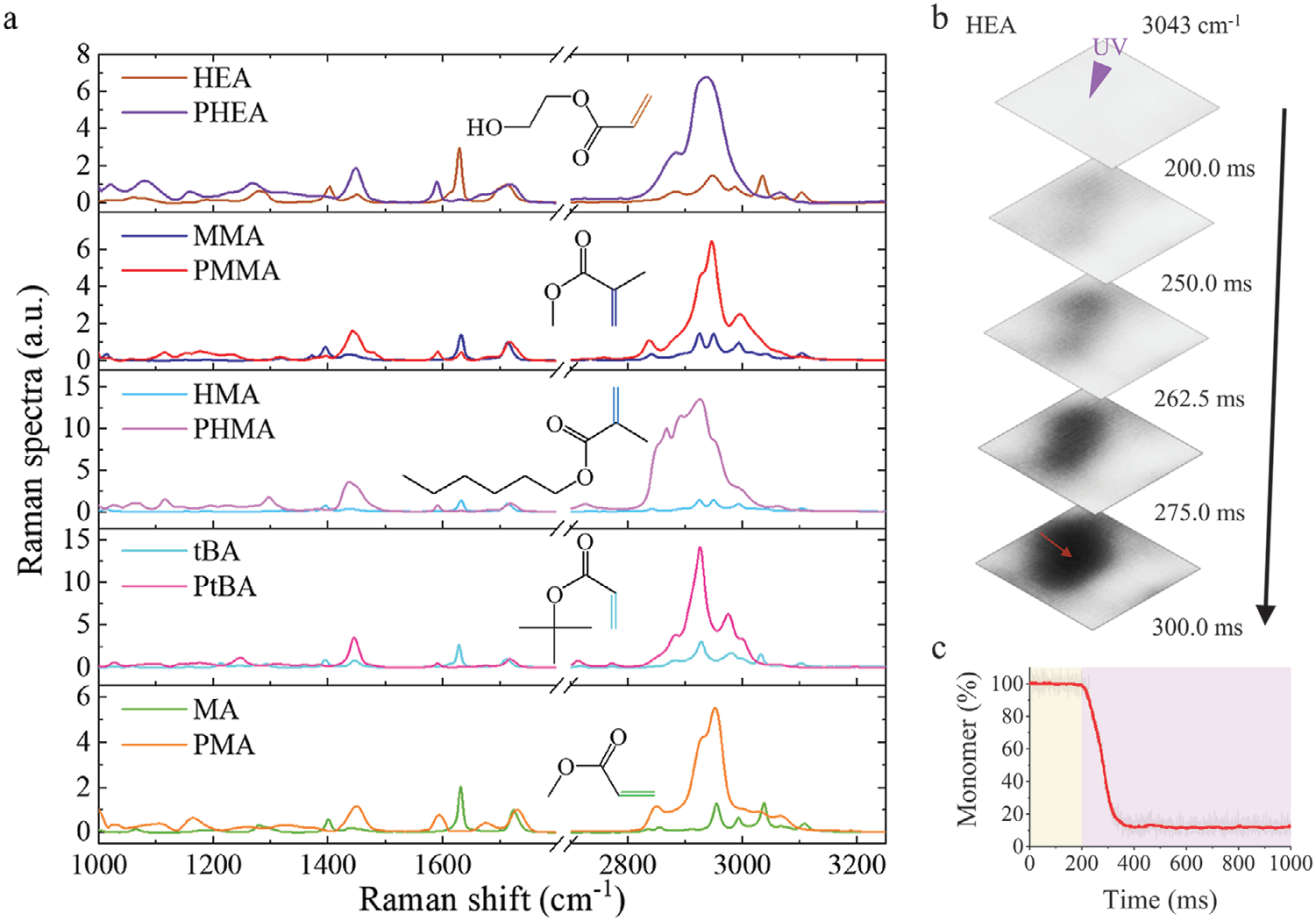
Raman spectra of other materials and faster polymerization process of HEA. a) The spontaneous Raman spectra of HEA, MMA, HMA, tBA, and MA and their corresponding polymers. All spectra were normalized by C=O Raman peak at 1720 cm^‐1^. b) Ultrafast imaging of polymerization process of HEA. c) Polymerization process on the indicated location in panel (b).

Photopolymerization is the basis of forming a variety of important materials, including gels, plastics, glues, and others. A significant amount of them are polymerized through chain reaction of C=C bonds in their monomers, such as 2‐hydroxyethyl acrylate (HEA), methyl methacrylate (MMA), hexyl methacrylate (HMA), *tert*‐butyl acrylate (tBA), methyl acrylate (MA), and so on. To validate that SRS imaging system is applicable to all these types of materials, we further examined the Raman spectra of these monomers and their polymers as shown in **Figure** **5**a. Except for the intensity and spectral differences that appeared between monomers and polymers in C—H region, we also found that the Raman band of C=C bond at ≈1630 cm^‐1^ in monomers vanished in the corresponding polymers. Therefore, COMB‐SRS microscopy is able to characterize the polymerization process of C=C bond‐related materials or others. It is worthy to note that only ≈80% MMA can convert to polymer at room temperature,^[^ ^24^ ^]^ thus we observed weak residue of C=C band in Raman of PMMA. We further performed ultrafast SRS imaging of HEA, which is known for much faster polymerization process (Figure 5b,c). On the initiation center, 80% of monomers were polymerized in less than 100 ms.

## 3 Conclusion

In summary, we developed ultrafast COMB‐SRS microscope to achieve direct imaging and quantification of polymerization kinetics in situ. Compared to the traditional tools, analytical tools to monitor chemical reaction, SRS microscopy allows noninvasive imaging of polymerization process with submicron spatial resolution and sub‐millisecond temporal resolution. By COMB‐SRS imaging, we present the polymer wave for the first time and discovered that the polymer wave actually stemmed from UV initiation and is driven by radicals persistently generated in the initiation center. To further improve the FOV and pixel number of the microscope, we can input 100 or more RF frequencies into AODs to simultaneously generate more Gaussian laser beams for parallel scanning. Figure S13, Supporting Information, shows the image of 100 laser focuses produced by AOD. Thus, the upper limit of the pixel number will be the total laser power that can be supplied for those beams. In addition, more modules of lock‐in‐free circuits will be required for independent detection of those more laser beams. By chemically seeing the polymerization process, COMB‐SRS with sufficient spatial and temporal resolution offers new possibilities for developing novel functional polymers, degradable plastics, controllable photoresist, and other advanced materials for wide applications in energy, material, life sciences, and other fields.

## 4 Experimental Section

##### Hyperspectral SRS and COMB‐SRS Microscopy

The SRS imaging system is based on a dual‐output femtosecond laser (InSight DeepSee, Spectra‐Physics, Newport), which provides two phase‐locked femtosecond lasers with a repetition rate of 80 MHz. The 220 fs output laser at wavelength of 1040 nm was modulated by a resonant electro‐optical modulator (EO‐AM‐R‐C2, Thorlabs) at 10.5 MHz with modulation depth of about 95%. After the pump (791 nm) and Stokes beams were spatially overlapped by a dichroic mirror (DMSP1000L, Thorlabs), two lasers were linearly chirped to ≈3 ps by 64 cm long SF57 glass rod. By tuning the relative time delay between them, Raman wavenumbers were shifted for spectral focusing‐based hyperspectral SRS imaging.

For COMB‐SRS imaging system, the pump and Stokes beams were deflected to 46 Gaussian beams by two AODs (DTSX‐A15‐900, AA SA), which were fed with two combs of radio frequencies produced by a 1GS/s arbitrary waveform generator (DG5252, RIGOL). Importantly, both waveforms were phase engineered carefully to minimize the peak‐to‐average power ratio before input to AODs.^[^ ^25^ ^]^ More details were presented by publication from Jalali and co‐workers.^[20a]^ Since the AOD crystals can seriously distort the laser beam to elliptical shape and broaden the pulse width, the spatial and temporal dispersion was compensated by two prisms (PS851, Thorlabs; BRP‐H‐ZF13‐30‐30‐65.5‐2, Union Optic) with incident angles of 62° and 72° for pump and Stokes lasers, respectively. After the correction, all deflected laser beams maintained pulse width of about 300 fs and good Gaussian shape for COMB‐SRS imaging. The combined 46‐ch laser combs were further scanned by a galvanometer (6210HSM40B, Cambridge Technology) and focused on the sample by a 60× water immersion objective (NA 1.2, UPLSAPO 60XW, Olympus). The pump laser was collected by another objective and restored to stationary laser comb by a compensate scanner. Before the pump laser beams were detected by a 46‐ch photodiode array (S4114‐46Q, Hamamatsu) and LIFCA, two shortpass filters (ET980SP, Chroma) and a bandpass filter (ZET820/200, Chroma) were installed to block the Stokes beams and UV laser. To generate UV laser for initiation of photopolymerization, a BBO crystal (4 × 4 × 0.6 mm, SHG@750‐1100 nm, CASTECH) was implemented to double the frequency of 791 nm laser to 396 nm. A single‐axis Galvanometer (GVS001, Thorlabs) and a slit (VA100, Thorlabs) were used to turn on and off the UV laser with <0.5 ms resolution, which was monitored by a home‐built fast photodiode.

##### Image Acquisition and Optical Settings

A 46‐ch LIFCA was built at 10.5 MHz (Figure S10, Supporting Information) to extract the weak SRS signals, and acquired images at the speed of 2000 frames per second by three 16‐ch acquisition cards (1 MHz per channel) (USB 2891, ART technology). For all SRS imaging experiments, the laser power was measured before objective (≈70% transmission), and the dwell time was set at 10 µs. As shown in Figure 1c,d, a UV lamp (≈360 nm, ZF‐5, Jiapeng) with illuminance of 0.44 mW cm^−2^ was employed to induce polymerization. During hyperspectral SRS imaging, the laser power of pump (791 nm) and Stokes beams were set to 60 and 160 mW, respectively. The field of view (FOV) was 50 × 50 µm^2^ with 50 × 50 pixels. In Figure 2a, a 5 W 395 nm light‐emitting diode (LED) was applied with intensity of 55 mW cm^−2^ to irradiate the acrylamide droplet for 3 min, and the laser power of pump and Stokes beams were 35 and 135 mW, respectively. The FOV was 200 × 200 µm^2^ with 200 × 200 pixels. In Figure 2c, 10 mW 396 nm UV laser was applied for 30 s before SRS imaging with laser power of 50 mW for pump (791 nm) and 100 mW for Stokes. The FOV was 120 × 120 µm^2^ with 600 × 600 pixels. For 3D SRS imaging (Figure 2d), the 10 mW UV laser was applied to the sample for 120 s, and the laser power of pump (795 nm) and Stokes beams was 30 and 80 mW, respectively. The FOV was 200 × 200 × 110 µm^3^ with 600 × 600 × 110 pixels. In Figure 2e, the laser power of pump (791 nm) and Stokes beams was 50 and 100 mW, respectively. The FOV is 100 × 100 µm^2^ with 400 × 400 pixels. For imaging of HEA in Figure 5b, 16 mW 396 nm laser was employed for polymerization initiation.

##### Sample Preparation

To prepare hydrogel solution, 1.6 g acrylamide (80001326, Sinopharm) was dissolved in 1 mL deionized water in 37 °C water bath for 10 min, and then 0.2 g Irgacure 2959 (B2959, BASF) and 0.2 g *N*,*N*′‐methylenebisacrylamide (30117826, Sinopharm) were added to the solution for additional 20 min. Before SRS imaging, a drop of hydrogel solution was sealed between two cover glasses (48393‐172, VWR). Monomers including HEA (H810915, Macklin), MMA (M813513, Macklin), HMA (H102084, Aladdin), tBA (B802797, Macklin), and MA (M812690, Macklin) were blended with 2% 2‐hydroxy‐2‐methylpropiophenone (H811172, Macklin), which was served as initiator. These solutions were polymerized by a 365 nm LED light source with intensity of about 12 mW cm^−2^.

##### Raman Experiments

All Raman spectra were obtained by a confocal Raman microscope (LabRAM HR800, Horiba Jobin Yvon) at room temperature with an integration time of 20 s. A 532 nm laser was focused to the samples by a 50× air objective (LMPlanFL, 0.75 NA, Olympus) with 5 mW on the sample.

## Conflict of Interest

The authors declare no conflict of interest.

## Author Contributions

H.L. built the hyperspectral SRS system and all electronic circuits. H.L., Y.C., H.T., Y.B., and S.G. carried out the construction of the COMB‐SRS imaging system. J.‐X.C. supported on resonant circuits. H.L. performed experiments. H.L. and P.W. analyzed the data. H.L., P.W., C.X., and J.‐X.C. wrote the manuscript with input from all authors. P.W. and H.L. conceived the concept. P.W. and J.‐X.C. supervised the project.

## Supporting information

Supporting Information

Supporting Movie 1

Supporting Movie 2

Supporting Movie 3

Supporting Movie 4
