# Supplementary material for: Imaging Chemical Kinetics of Radical Polymerization with an Ultrafast Coherent Raman Microscope
Source: Adv Sci (Weinh). 2020 Mar 9;7(10):1903644. doi: 10.1002/advs.201903644 (PMC7237838; doi:10.1002/advs.201903644)
Supplement: Supplementary file 1 — Supporting Information [file ADVS-7-1903644-s001.pdf]

Copyright WILEY-VCH Verlag GmbH & Co. KGaA, 69469 Weinheim, Germany, 2020.

ADVANCED SCIENCE
Open Access

Supporting Information

for *Adv. Sci.,* DOI: 10.1002/advs.201903644

Imaging Chemical Kinetics of Radical Polymerization with an Ultrafast Coherent Raman Microscope

Haozheng Li, Yong Cheng, Huajun Tang, Yali Bi, Yage Chen, Guang Yang, Shoujing Guo, Sidan Tian, Jiangshan Liao, Xiaohua Lv, Shaoqun Zeng, Mingqiang Zhu, Chenjie Xu, Ji-Xin Cheng, and Ping Wang**

Supporting Information

Imaging Chemical Kinetics of Radical Polymerization with an Ultrafast Coherent Raman Microscope

Haozheng Li, Yong Cheng, Huajun Tang, Yali Bi, Yage Chen, Guang Yang, Shoujing Guo, Sidan Tian, Jiangshan Liao, Xiaohua Lv, Shaoqun Zeng, Mingqiang Zhu, Chenjie Xu, Ji-Xin Cheng & Ping Wang**

H. Li, Dr. Y. Cheng, H. Tang, Y. Bi, Y. Chen, G. Yang, S. Guo, S. Tian, J. Liao, Prof. X. Lv, Prof. S. Zeng, Prof. M. Zhu, Prof. P. Wang
Britton Chance Center for Biomedical Photonics, Wuhan National Laboratory for Optoelectronics-Huazhong University of Science and Technology
Wuhan, Hubei 430074, China
MoE Key Laboratory for Biomedical Photonics, Collaborative Innovation Center for Biomedical Engineering, School of Engineering Sciences, Huazhong University of Science and Technology
Wuhan, Hubei 430074, China
E-mail: p_wang@hust.edu.cn

Prof. J.-X. Cheng
Department of Biomedical Engineering, Boston University
Boston, Massachusetts 02215, United States
E-mail: jxcheng@bu.edu

Prof. C. Xu
School of Chemical and Biomedical Engineering, Nanyang Technological University
Singapore 637457, Singapore

Contents

1. Supplementary figures 3

1.1. Photolysis and initiating radical formation..... 3

1.2. Schematic of the spectral-focusing based SRS imaging system. 3

1.3. Shot noise limit detection at 10.5 MHz.. 4

1.4. Noninvasive SRS imaging of acrylamide solution. 4

1.5. The calculated conversion efficiency of hydrogel droplet in Figure 2a. 5

1.6. The corresponding conversion map of Figure 2c. 5

1.7. Repeated experiment of Figure 2c. 6

1.8. Picture of AOD system of COMB-SRS. 6

1.9. Compensation for spatial dispersion by prism. 7

1.10. Detection circuits for COMB-SRS microscope. 7

1.11. Image processing steps of COMB-SRS images. 8

1.12. COMB-SRS imaging of polymerization process with manipulated UV imitation. 9

1.13. Image of 100 laser focuses generated by AOD. 9

2. Supplementary notes 10

2.1. Principles of AOD. 10

2.2. Dispersion compensation. 11

2.3. Circuits design for LIFCA. 12

3. Supplementary videos 13

3.1. Two-species 3D SRS imaging of monomer and formed polymer structure. 13

3.2. SRS imaging of polymerization dynamics as the UV laser was set to 1, 3, 5, 9 mW. 13

3.3. Label-free imaging of polymerization initiation with speed of 2000 frame/second. 13

3.4. Mapping the ultrafast dynamics of polymerization rate. 13

References: 14

1. Supplementary figures

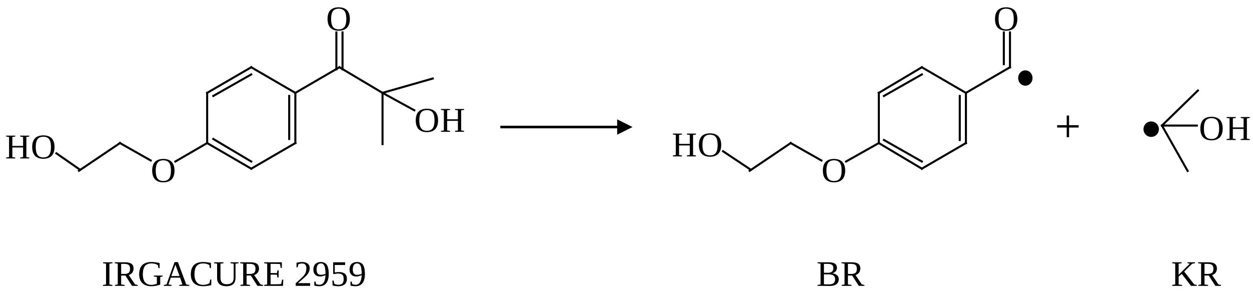

Figure S1. Photolysis and initiating radical formation. IRGACURE 2959, benzoyl radical (BR), ketyl radical (KR).[1]

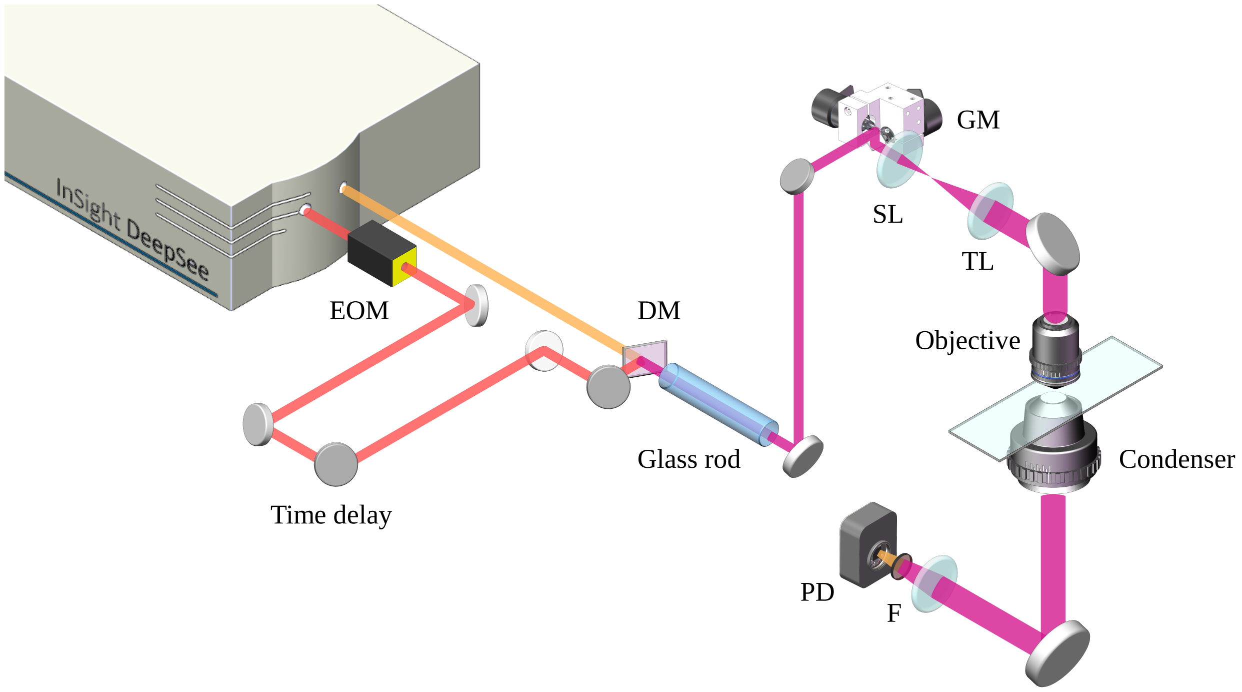

Figure S2. Schematic of the spectral-focusing based SRS imaging system. EOM: electro-optic modulator; DM: dichroic mirror; GM: galvanometer; SL: scanning lens; TL: tube lens; F: optical filters; PD: photodiode.

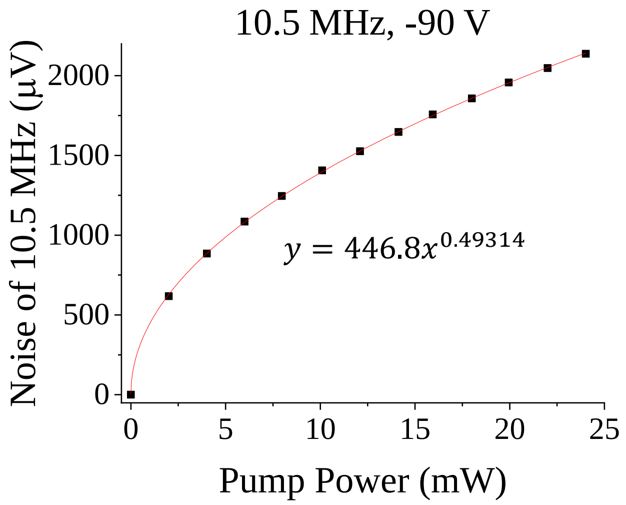

Figure S3. Shot noise limit detection at 10.5 MHz. Pump laser was detected at 10.5 MHz. The reverse voltage applied to PD was 90 V to avoid laser power saturation.

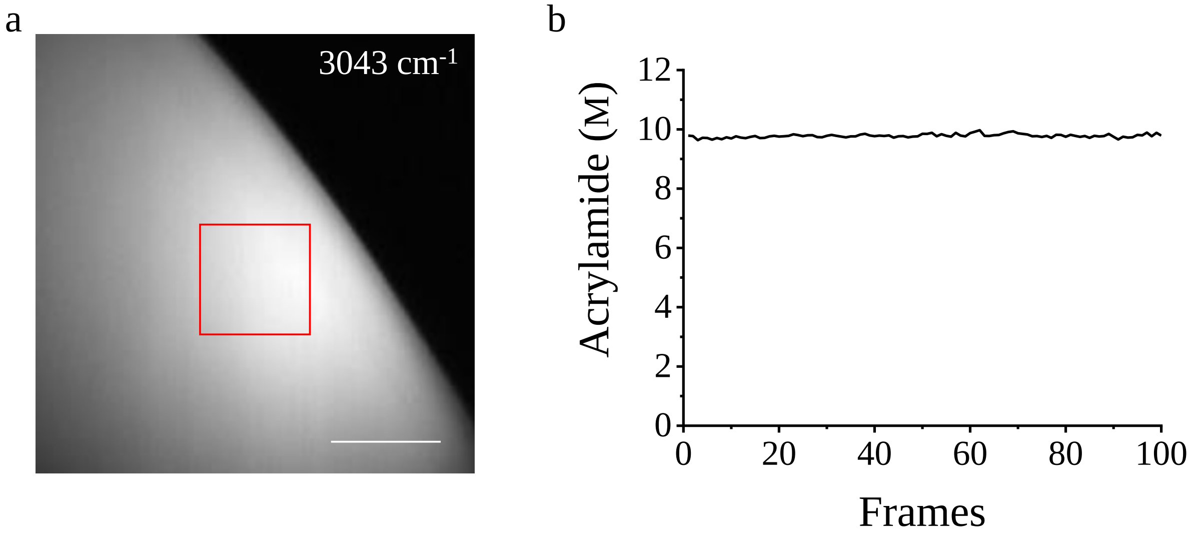

Figure S4. Noninvasive SRS imaging of acrylamide solution. a) SRS image of hydrogel solution with IRGACURE 2959. b) SRS intensity (in the red box of (a)) kept constant under continuous SRS laser scanning. NIR SRS lasers have not induced or interfered the polymerization process. Scale bar, 50 μm.

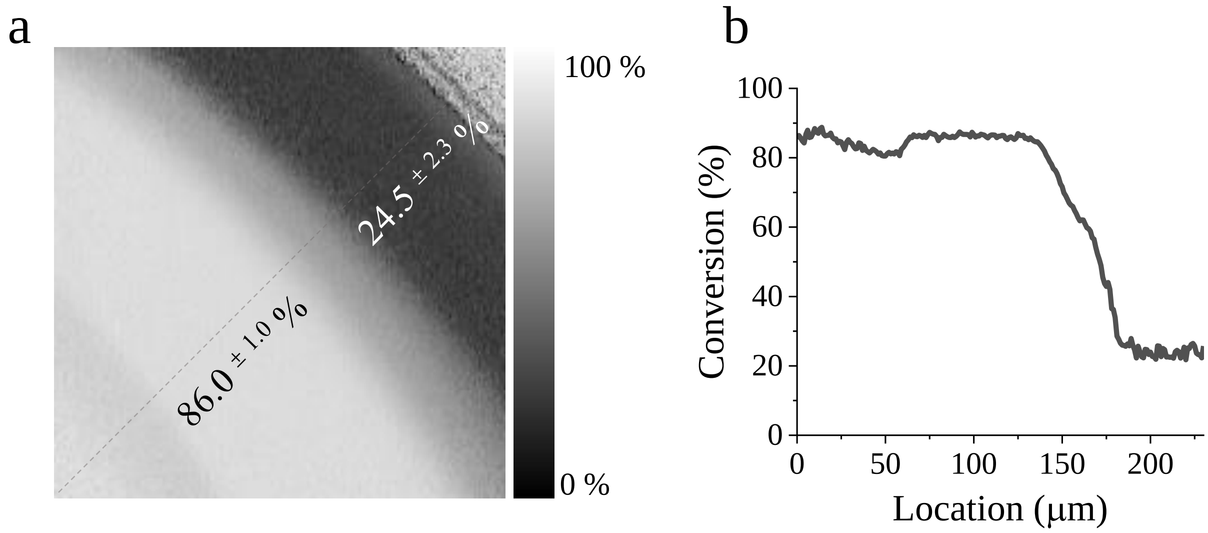

Figure S5. The calculated conversion efficiency of hydrogel droplet in Figure 2a. a) The corresponding conversion map. Conversion map was obtained by image normalization, in which the SRS image of polymer was divided by the sum of SRS images representing polymer (2928 cm^{-1}) and monomer (3043 cm^{-1}). b) The intensity profile along the dashed line in (a).

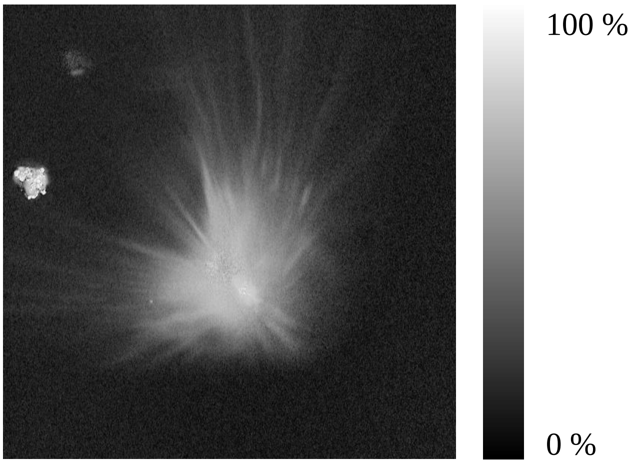

Figure S6. The corresponding conversion map of Figure 2c.

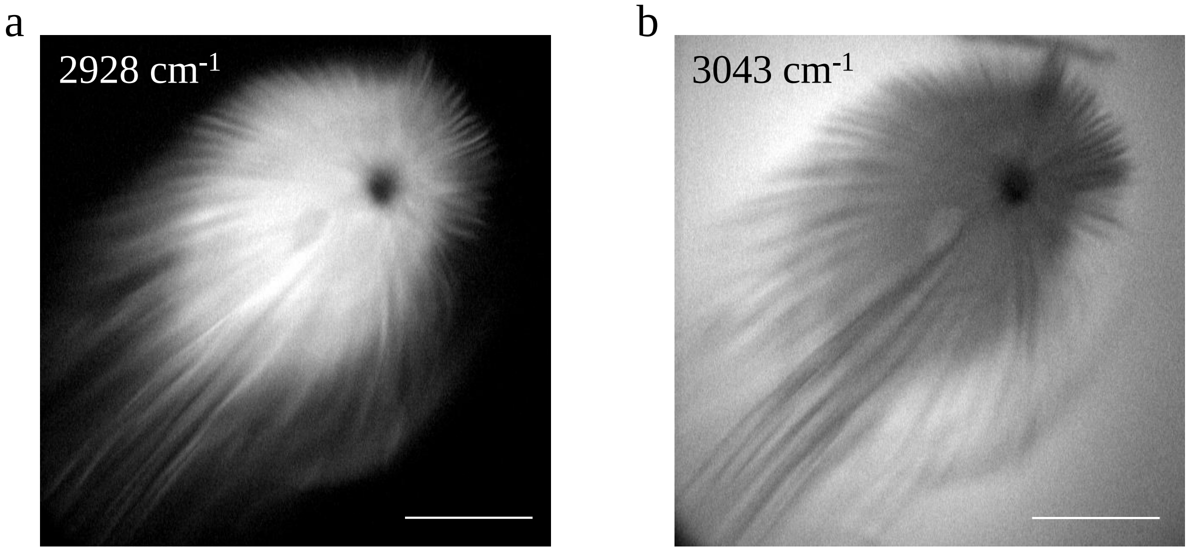

Figure S7. Repeated experiment of Figure 2c. SRS images at 2928 cm^{-1} and 3043 cm^{-1}. Scale bars, 50 μm.

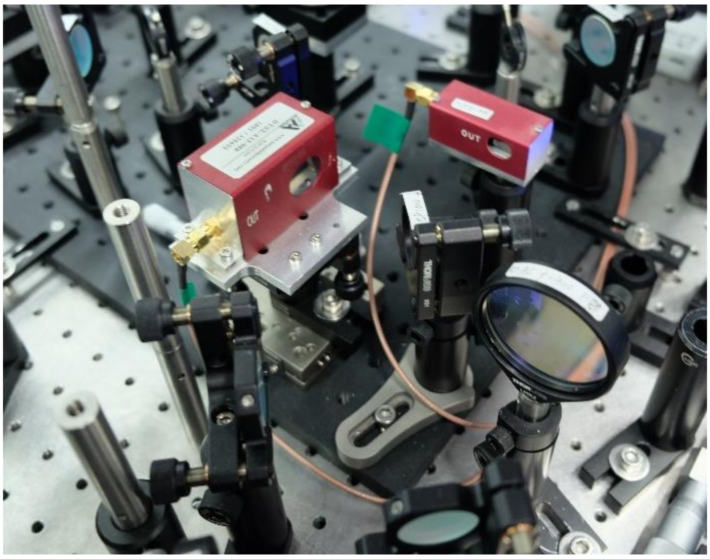

Figure S8. Picture of AOD system of COMB-SRS.

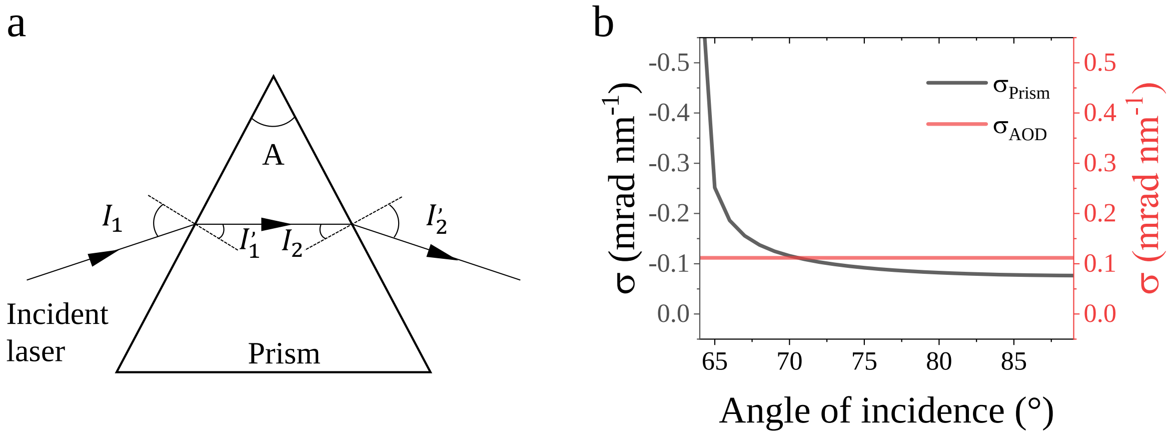

Figure S9. Compensation for spatial dispersion by prism. a) Diagram of laser refraction in prism. b) Spatial dispersion of prism correlated with incidence angle of the laser.

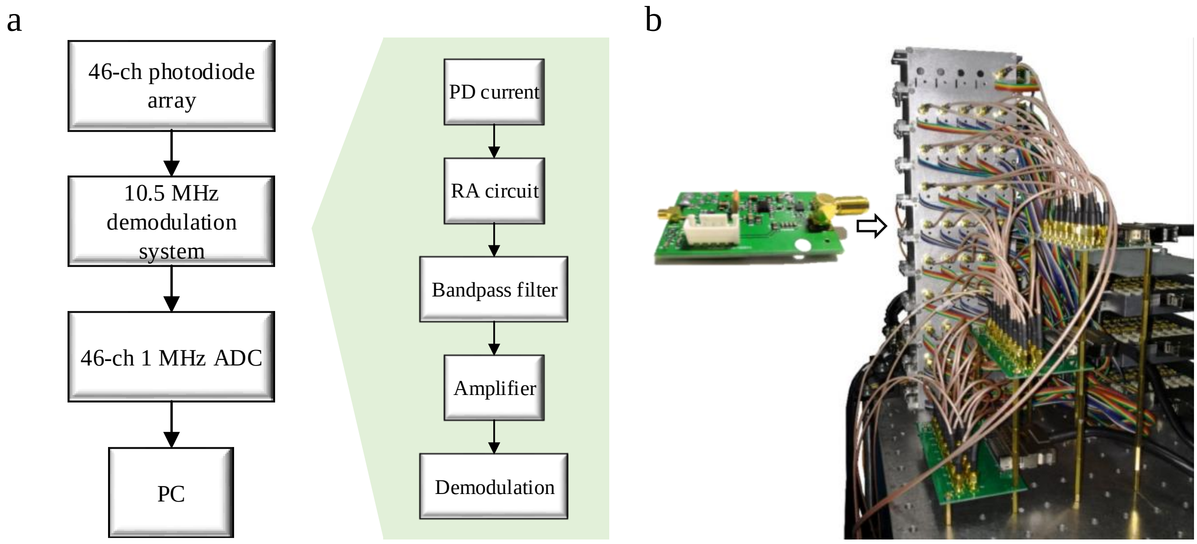

Figure S10. Detection circuits for COMB-SRS microscope. a) 46-ch SRS signals were extracted by RA circuits and filtered by bandpass filters. The AC signals were amplified again and demodulated by lock-in free circuits. b) The picture of actual circuits system with 46 independent demodulation units.

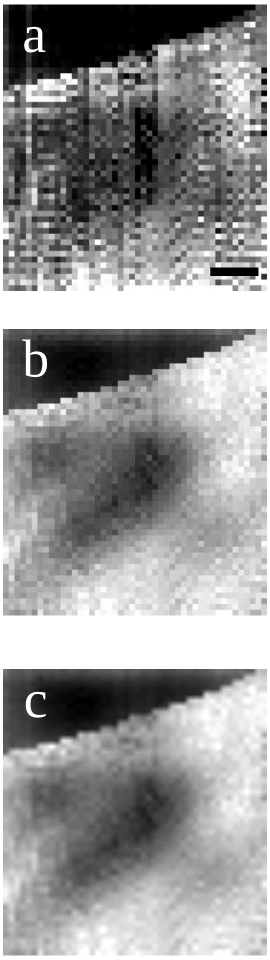

Figure S11. Image processing steps of COMB-SRS images. a) Raw data. b) Image after frequency domain filter (2D Fourier transformation). c) Image after bilinear interpolation. Scale bar, 5 μm.

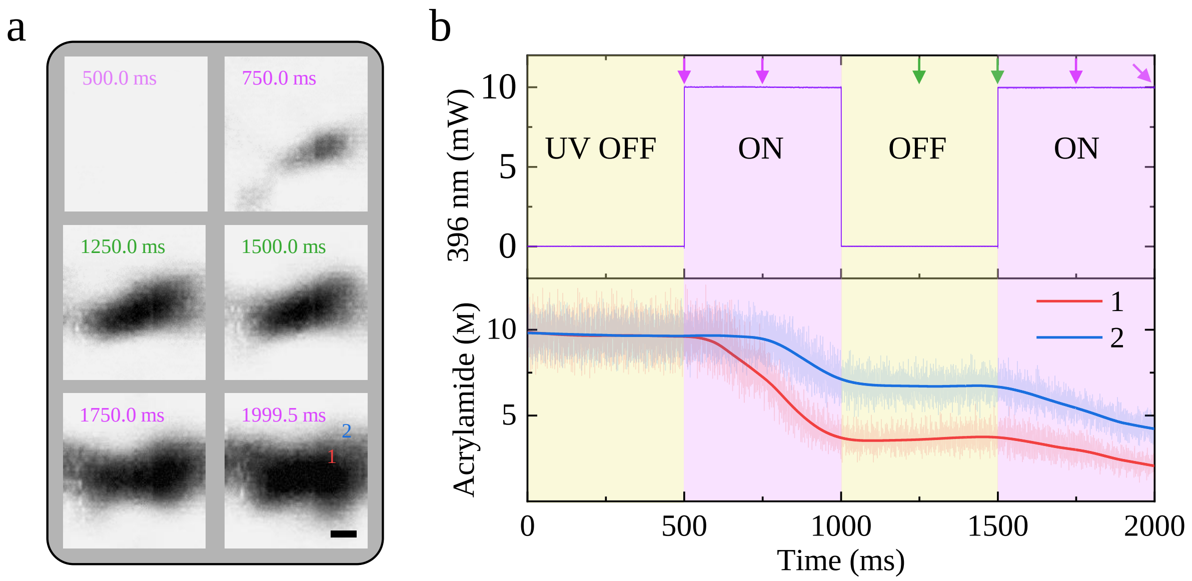

Figure S12. COMB-SRS imaging of polymerization process with manipulated UV imitation. a) Images of polymerization process with time. Scale bar, 5 μm. b) Concentration change on the indicated locations in (a). The arrows with color indicate the time of SRS images shown in (a).

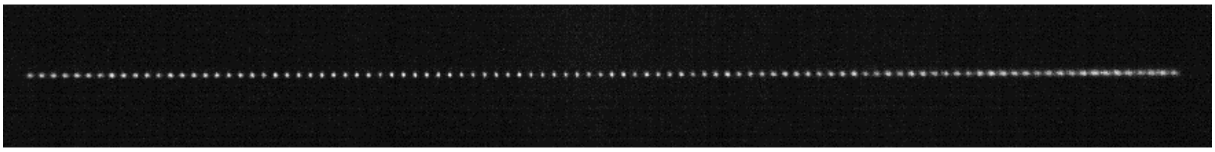

Figure S13. Image of 100 laser focuses generated by AOD.

2. Supplementary notes

Note S1. Principles of AOD.

When the incident angle of the input laser meets the Bragg condition of AOD, the laser will be deflected to the first-order with maximum efficiency:

$$\theta_{Bragg} = \frac{\lambda f}{2v} \tag{1}$$

Where θ_{Bragg} is the Bragg diffraction angle, λ is the wavelength of the incident laser, f is the driving RF frequency of AOD, and v is the propagation speed of sound wave in the crystal.

The deflection angle of the laser beam is θ_{SET}:

$$\theta_{SET} = \frac{\lambda f}{v} \tag{2}$$

To produce 46 laser beams with different deflection angles, we implemented a function generator to input 46 RF frequencies to the AOD.

Note S2. Dispersion compensation.

AOD can induce positive spatial and temporal dispersion to incident laser, and degrade the resolution and sensitivity of SRS imaging.[2] Thus, we applied prisms to introduce negative dispersion to correct the distortions on the deflected lasers. From Eq. (2), we can determine the spatial dispersion constant σ_A of AOD to be $\sigma_A = f/v$. Here, v is the sound velocity about 677 m/s, and we selected the center frequency f of AOD to be 75.5 MHz. Thus, the angular dispersion constant introduced by AOD is about 0.112 mrad/nm.

The spatial dispersion constant of a prism can be calculated by：

$$\sigma_{\mathrm{P}} = \frac{\cos I_2 \tan I'_1 + \sin I_2}{\cos I'_2} \times \frac{\mathrm{dn}}{\mathrm{d}\lambda} \quad (3)$$

Here, the angles (I) were illustrated in Figure S9a. In our experiment, we chose SF11 as material of the prism. The refractive index of SF11 is 1.765, and the $\frac{\mathrm{dn}}{\mathrm{d}\lambda}$ for this material is about -0.04924$\mu\mathrm{m}^{-1}$. By Eq. (3), we can plot the correlation between dispersion constant of the prism and the angle of laser incidence (Figure S9b). Thus, we determined the apex angle of the isosceles prism and the angle of incidence to be 64.5°and 72°respectively. Furthermore, we adjusted the distance between the prism and the AOD to compensate the temporal dispersion with an autocorrelator (Carpe, APE).

Note S3. Circuits design for LIFCA.

The 46-channel photodiode was used to detect 46 pump beams in COMB-SRS. The weak photo currents generated by photodiode array were amplified by 46-channel resonant amplifiers and lock-in free circuits.[3] Each unit of LIFCA includes independent resonant amplifier, 10.5 MHz band pass filter, two amplifiers and demodulation module. The 46-channel SRS signals were then acquired simultaneously by three acquisition cards.

3. Supplementary videos

Video S1. Two-species 3D SRS imaging of monomer and formed polymer structure (corresponding to Figure 2d).

Video S2. SRS imaging of polymerization dynamics as the UV laser was set to 1, 3, 5, 9 mW.

Video S3. Label-free imaging of polymerization initiation with speed of 2000 frame/second (corresponding to Figure 4a).

Video S4. Mapping the ultrafast dynamics of polymerization rate (corresponding to Figure 4c).

References:

[1] M. Liu, M.-D. Li, J. Xue, D. L. Phillips, *J. Phys. Chem. A* **2014**, *118*, 8701-8707.
[2] R. H. Jiang, Z. Q. Zhou, X. H. Lv, S. Q. Zeng, *Rev. Sci. Instrum.* **2012**, *83*, 043709.
[3] C. S. Liao, M. N. Slipchenko, P. Wang, J. J. Li, S. Y. Lee, R. A. Oglesbee, J. X. Cheng, *Light Sci. Appl.* **2015**, *4*, e265.
